# Barrier Capacity of Human Placenta for Nanosized Materials

**DOI:** 10.1289/ehp.0901200

**Published:** 2009-11-12

**Authors:** Peter Wick, Antoine Malek, Pius Manser, Danielle Meili, Xenia Maeder-Althaus, Liliane Diener, Pierre-Andre Diener, Andreas Zisch, Harald F. Krug, Ursula von Mandach

**Affiliations:** 1 Empa, Swiss Federal Laboratories for Material Testing and Research, Laboratory for Materials–Biology Interactions, St. Gallen, Switzerland; 2 University Hospital Zurich, Department of Obstetrics, Zurich, Switzerland; 3 Institute of Pathology, Cantonal Hospital, St. Gallen, Switzerland

**Keywords:** barrier tissue, ex vivo perfusion, human placenta, nanoparticles, nanotoxicity

## Abstract

**Background:**

Humans have been exposed to fine and ultrafine particles throughout their history. Since the Industrial Revolution, sources, doses, and types of nanoparticles have changed dramatically. In the last decade, the rapidly developing field of nanotechnology has led to an increase of engineered nanoparticles with novel physical and chemical properties. Regardless of whether this exposure is unintended or not, a careful assessment of possible adverse effects is needed. A large number of projects have been carried out to assess the consequences of combustion-derived or engineered nanoparticle exposure on human health. In recent years there has been a growing concern about the possible health influence of exposure to air pollutants during pregnancy, hence an implicit concern about potential risk for nanoparticle exposure *in utero*. Previous work has not addressed the question of whether nanoparticles may cross the placenta.

**Objective:**

In this study we investigated whether particles can cross the placental barrier and affect the fetus.

**Methods:**

We used the *ex vivo* human placental perfusion model to investigate whether nanoparticles can cross this barrier and whether this process is size dependent. Fluorescently labeled polystyrene beads with diameters of 50, 80, 240, and 500 nm were chosen as model particles.

**Results:**

We showed that fluorescent polystyrene particles with diameter up to 240 nm were taken up by the placenta and were able to cross the placental barrier without affecting the viability of the placental explant.

**Conclusions:**

The findings suggest that nanomaterials have the potential for transplacental transfer and underscore the need for further nanotoxicologic studies on this important organ system.

Humans have always been exposed to airborne particles through natural sources such as volcanoes, forest fires, or desert dust. After the Industrial Revolution in the 19th century, emission of anthropogenic particles into the atmosphere significantly increased. This increased exposure to respirable fine particles (< 2.5 μm in diameter) and ultrafine particles (UFPs; < 0.1 μm in diameter). Epidemiologic and *in vivo* studies have shown potential toxic effects of fine particles and UFPs on human health (Kreyling et al. 2004; Oberdörster and Utell 2002). When inhaled, UFPs are deposited in the respiratory tract and may provoke inflammation or granuloma formation in the lung and induce systemic effects such as prothrombotic responses (Nemmar et al. 2008) or cardiovascular changes (Alfaro-Moreno et al. 2007; Kreyling et al. 2004; Nemmar et al. 2007, 2008). Despite the presence of several highly efficient lung clearance mechanisms, different studies have shown that the transfer of nanoparticles from the lung to the blood is possible (Geiser et al. 2005; Nemmar et al. 2005; Rothen-Rutishauser et al. 2006).

With the broad use of nanotechnology, intentional production of nanoobjects in the size range of 1–100 nm has further increased the number and variety of nanoparticles to which humans can be exposed. New applications of engineered nanoparticles in nanomedicine such as nanoparticulate contrast agents (Rudin and Weissleder 2003), cancer treatments (Thiesen and Jordan 2008), and vaccines (Reddy et al. 2007) are in development that will be directly injected in the bloodstream of patients.

Most of the recent research dealing with potential health hazards of nanoparticles has focused on cells and tissues that are likely to come into immediate contact with airborne particles. There is a growing concern about the possible influence on health of the exposure to nanoparticles during pregnancy or early childhood (Lacasana et al. 2005). A recently performed cohort study suggests that prenatal exposure to air pollution might be associated with higher respiratory need and airway inflammation in newborns (Latzin et al. 2008). However, it remains unclear whether these effects were caused by particles crossing the placenta or by mediators induced in the maternal circulation such as proinflammatory factors.

First indications that nanosized materials may cross the placental tissue came from a recent study in rats, showing that nanosized gold was transferred to the embryos 24 hr after intravenous injection (Semmler-Behnke et al. 2007). However, these data cannot be extrapolated to humans because the anatomy and physiology of the human placenta are unique. One main difference is that in humans the syncytiotrophoblasts arise from the fusion of cytotrophoblast cells and form a true syncytium with no lateral cell membranes, whereas in rats or mice three trophoblast layers are present between maternal blood and fetal blood capillaries (for comprehensive reviews, see Enders and Blankenship 1999; Takata et al. 1997). Thus, the transport efficiency for xenobiotics or nanoparticles across the placenta has to be defined for humans explicitly.

In detail, the cellular barrier between the maternal and the fetal blood is formed by the syncytiotrophoblast layer, which faces the maternal environment, and the endothelial cell layer of the fetal microcapillaries. Between these two cell layers there are several stromal cells such as cytotrophoblasts, fibroblasts, and Hofbaur cells (placental macrophages). This layer is relatively thick in early pregnancy and becomes progressively thinner with gestational age. This reduction in thickness together with an increase in the number of fetal capillaries enhances the efficiency of maternal–fetal exchange during the development of the fetus (Enders and Blankenship 1999). The transfer of substances and the amount that enters the fetal circulation are tightly controlled by at least four different mechanisms: simple diffusion, active transport, biotransformation through metabolic enzymes, and phagocytosis or pinocytosis (Syme et al. 2004). The plasma membrane of the syncytiotrophoblast is polarized, consisting of the brush border membrane that is in direct contact with maternal blood and the basal membrane that faces the fetal circulation (Ganapathy et al. 2000). The two membranes are further distinguishable from each other by their protein, receptor, and transporter composition. The brush border membrane contains specific transporters, such as those for serotonin (SER transporter) or carnitine (novel organic cation transporter OCTN2), or unspecific transporters such as P-glycoprotein, a member of the multidrug-resistance gene family. The folate transporter FOLT1, together with the folate receptor of the brush boarder membrane, is typically expressed in basal membrane and mediates the transport of vitamins across the placenta (Ganapathy et al. 2000).

The dual recirculation human placental perfusion model (Panigel et al. 1967; Schneider et al. 1972) provides a controlled system for studying the transplacental transport of various substances and is commonly used for pharmacokinetic studies. In the present study we used this model to investigate whether nanoparticles can cross the placental barrier and whether this process is size dependent. We chose fluorescently labeled polystyrene (PS) beads with diameters of 50, 80, 240, or 500 nm as model particles. In addition, we assessed the viability and functionality of the placenta after particle perfusion.

## Materials and Methods

### Polystyrene beads

Fluorescent PS particles of 50 and 240 nm were commercially available from Kisker (PFP-00552 and PFP-0252; G. Kisker GbR, Steinfurt, Germany), and 80- and 500-nm beads were purchased from Polyscience (catalog nos. 17150 and 17152; Polyscience Europe GmbH, Eppelheim, Germany). The particle stock solution was vortexed 15 min before each perfusion assay.

### Characterization of PS bead suspension

We used a scanning electron microscope (SEM; S-4800, Hitachi High-Technologies Europe GmbH, Krefeld, Germany) to analyze the visual appearance of the as-received PS beads. We measured the size distribution of the PS beads in perfusion medium with a NanoSight LM 20 System (NanoSight Ltd., Amesbury, Wiltshire, UK) at a particle concentration of about 10^6^ to 10^9^ particles/mL and are presented as size–density plots. The density plot of the perfusion medium without beads has been reduced by a factor of 5 compared with particle measurement in relation to the number of counting events measured.

We tested the stability of the fluorescent PS beads in different solutions, including the perfusion medium. Briefly, the beads were diluted with phosphate-buffered saline (pH 4), 1% sodium dodecyl sulfate, 100% ethanol, and perfusion medium to a concentration of 10 μg/mL and incubated for 24 hr at 37°C. The fluorescence of 100 μL suspension was measured before and after filtration with a 0.1-μm Pall Acrodisc hydrophilic polyethersulfone filter (Pall Corporation, Port Washington, NY, USA), using a microplate reader (BioTex FLx800; Witec AG, Littau LU, Switzerland) with excitation and emission wavelengths of 485 and 528 nm, respectively.

We determined the detection limit of PS beads for each type of particles separately by serial dilution in perfusion medium in the range of 0.02–10 μg/mL. The detection limit was defined as the lowest concentration that showed a significant increase in the fluorescent intensity compared with pure perfusion medium.

We measured zeta potentials of different PS bead suspensions using a Malvern zetameter (Malvern Instruments Ltd, Malvern, Worcestershire, UK). First, PS beads were dispersed in water to a concentration of 1 mg/mL by vortexing for 10 min. A second suspension was obtained by vortexing the PS beads in perfusion medium for 10 min.

### *Ex vivo* dual recirculating human placental perfusion model

**The placental perfu**sion technique was originally developed by Panigel et al. (1967) and was further improved by Schneider et al. (1972). The dual recirculating perfusion was performed according to Malek et al. (2009). Intact placentas were obtained from uncomplicated term pregnancies either after vaginal or cesarean delivery at the Department of Obstetrics of the University Hospital Zurich. All mothers gave written informed consent for the use of the placentas in this study. To minimize damage, the placentas were placed in 200 mL of a 0.9% sodium chloride solution preheated to 37°C, and then transported in a Styrofoam box to the research laboratory within 20 min of delivery. To perfuse the fetal side, the artery and vein of an intact cotyledon were cannulated. Subsequently, the placental tissue was fixed in a tissue holder and then placed into a perfusion chamber. The maternal side was perfused by introducing three blunt metal cannulas into the intervillous space by penetration of the decidual plate. Furthermore, a venous drain, connected with a peristaltic pump (Reglo Digital; Ismatec, Glattbrug, Switzerland) removed the perfusate from the chamber and returned it to the maternal reservoir. Fetal and maternal cannulas were connected to separate perfusion circuits. Peristaltic pumps held the fetal circuit flow at a rate of 6.0 mL/min and the maternal circuit at 12.0 mL/min.

The perfusion medium is composed of NCTC-135 tissue culture medium (ICN Biomedicals, Inc., Irvine, CA, USA) diluted with Earl’s buffer (1:1) supplemented with glucose (G 7528, 2 g/L; Sigma Switzerland, Buchs SG, Switzerland), dextran 40 (10 g/L; Sigma 31389), bovine serum albumin (8 g/L; Sigma), sodium heparin (2,500 IU/L; GlaxoSmithKline AG, Münchenbuchsee, Switzerland), and amoxicillin (Clamoxyl, 500 mg/L; GlaxoSmithKline). Two oxygenators (LSI-OX, Living Systems Instrumentation Inc., Burlington, NH, USA) were used, one with 95% N_2_ and 5% CO_2_ for the fetal circuit, and one with 95% air and 5% CO_2_ for the maternal perfusate. Both perfusates were maintained at a temperature of 37°C by a water bath (Glas water bath; UNI-GLAS, Zürich, Switzerland). The experiments included a 20-min period of open perfusion of both compartments (prephase) to flush the blood out of the intervillous space and the villous vascular compartment and to allow recovery of the placental tissue from the ischemic period after delivery. After the prephase, the maternal perfusate was exchanged with a perfusate containing PS beads of 50, 80, 240, or 500 nm, respectively, at a final concentration of 25 μg/mL and ^14^C-antipyrine (50 nCi/mL; specific activity, 4.7 mCi/mmol). The experiment was continued in closed maternal and fetal circuits with equal starting volumes of 120 mL. After 180 min the same amount of beads was added to the maternal circuit. The integrity of the fetal circulation was monitored by assessing the leak in the fetal-to-maternal direction, including the measurement of the volume loss of the fetal perfusate.

The criteria for a successful perfusion were *a*) visual control: intact membranes, no lesion, no disruption of the placenta; *b*) leak from fetal to maternal side < 4 mL/min (Malek et al. 1994); *c*) ^14^C-antipyrine values: equilibrium between maternal and fetal circuit after 4–6 hr (Challier 1985); *d*) fetal perfusion pressure < 70 mm Hg at all times; and *e*) fetal pH at physiologic range of 7.2–7.4. To determine the concentration of fluorescent PS particles in the maternal and fetal circuit, we removed 100 μL perfusion medium from each circuit at the indicated time points. After centrifugation at 800 rpm to remove residual erythrocytes, fluorescence was read at 485-nm excitation and 528-nm emission in a microplate reader (BioTex FLx800, Witec AG).

### Permeability and viability of the placental tissue

We evaluated the permeability of the placenta by monitoring the transplacental transfer of ^14^C-antipyrine as described previously (Challier 1985). Briefly, 50 nCi/mL ^14^C-antipyrine were added to the maternal circuit, and 300 μL of the perfusion medium from each reservoir was transferred into separate scintillation tubes and mixed with 3 mL scintillation cocktail (Irgasafe Plus Scintillation Cocktail, Zinsser Analytic, Frankfurt, Germany). ^14^C radioactivity was immediately measured for 5 min in a beta counter (Packard Tri-Carb 2200 liquid scintillation analyzer; GMI, Ramsey, MN, USA). Maintenance of placental function was assessed by measuring the production and accumulation of the two placental hormones leptin and human chorionic gonadotropin using an enzyme-linked immunosorbent assay as described previously (Malek et al. 2000, 2001). Briefly, the net production (NP) of human choriongonadotropin or leptin was estimated as NP = (end tissue content minus initial tissue content) plus accumulation. To determine the metabolic activity of the placenta, we used the sum of changes in total (maternal and fetal) content over time divided by the perfused cotyledon weight to calculate glucose consumption or lactate production, which were measured with an automated blood gas system (ABL800 FLEX automated benchtop analyzer; Radiometer Medical ApS, Copenhagen, Denmark).

### Detection of PS beads in the fetal circuit after perfusion

A 1.5-mL sample of the fetal perfusion medium was taken after 6 hr of perfusion and was centrifuged at 25,000 relative centrifugal force for 30 min at 4°C. This allowed the concentration of PS beads > 50 nm in the pellet, because no fluorescent signals were detected in the supernatant after centrifugation. The pellet was washed once with Millipore water, applied onto a carbon-coated copper grid (400 mesh; Pelco, Redding, CA, USA) and processed for transmission electron microscopy (TEM) analysis.

### Statistical analysis

Data are shown as mean ± SE from at least four independent experiments. Statistical analysis was made with the Mann–Whitney two-sample test and the Wilcoxon test. A *p*-value < 0.05 was considered to be statistically relevant.

## Results

### PS particle characterization

**Figure 1:**
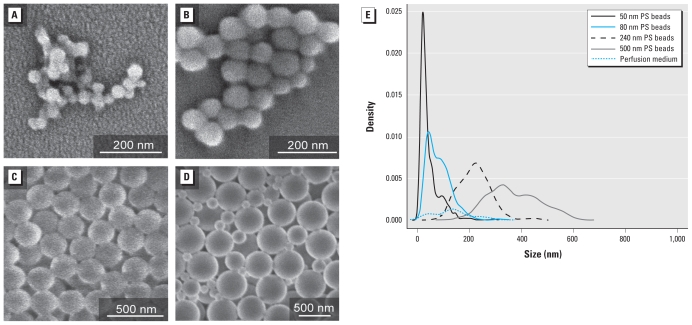
SEM and size distribution of PS beads. (*A*–*D*) SEM micrographs depict the beads with diameters of 50 nm (*A*), 80 nm (*B*), 240 nm (*C*), and 500 nm (*D*). (*E*) The size distributions of all applied particles were measured in the perfusion medium, presented as a size–density plot.

Table 1 summarizes details of particle characterization, such as particle size, surface area, and total surface charge in pure water and in perfusion medium. The zeta potential measurement of the PS beads in perfusion medium resulted in slightly negative values ranging from −12.8 ± 1.20 to −6.23 ± 0.20 mV, indicating that the albumin stabilizes the spheres in the perfusion medium. Size distribution analysis confirmed that suspensions of 50-nm, 80-nm, and 240-nm beads were relatively monodisperse (Figure 1A–C, E). However, we observed particles of various sizes in SEM images as well as in size–density plots of 500-nm suspensions (Figure 1D, E).

The use of fluorescent nanoparticles, especially of PS beads, has been shown to cause experimental problems because of a possible transfer of the lipophilic dye to the surrounding tissue and/or cells (Kanno et al. 2007; Pietzonka et al. 2002). To exclude false-positive results, we measured the stability of the fluorescent dye in different solutions, including our perfusion medium. Except for pure ethanol, where we detected a release of 33.7 ± 0.74% of the fluorescent dye for 240-nm beads and 35.9 ± 10.2% for 500-nm beads, the fluorescence remained stable in all tested conditions, including our perfusion medium (data not shown).

We experimentally determined the detection limit of the fluorescent PS particles in perfusion medium for each particle size. The minimum concentration of particles that still allowed the detection of a significant fluorescent signal was 1.25 μg/mL for 50-nm, 1.64 μg/mL for 80-nm, 0.63 μg/mL for 240-nm, and 0.21 μg/mL for 500-nm beads (Table 1).

### Perfusion profiles of PS beads of different sizes

**Figure 2:**
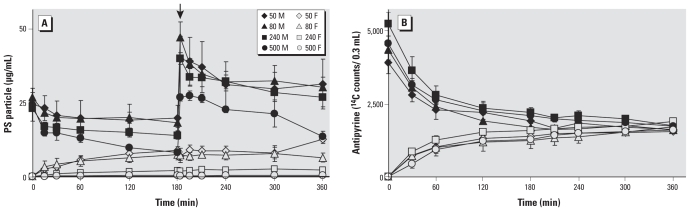
Perfusion profiles of PS beads and ^14^C-antipyrine. After the prephase, PS beads of different sizes were added to the maternal circuit to a final concentration of 25 μg/mL and the amount of ^14^C-antipyrine and PS beads in both maternal circuits (M, solid symbols) and fetal circuits (F, open symbols) were measured after the indicated time points. (*A*) In the fetal circuit, significantly increased levels of 50-, 80-, and 240-nm PS beads were measured after only a few minutes of perfusion, whereas the 500-nm beads were retained in the placental tissue and maternal circuit. A second addition of particles after 180 min (arrow) did not further increase the amount of beads in the fetal circuit. (*B*) The perfusion profiles of ^14^C-antipyrine were not affected by the presence of beads and maintained equilibrium until the end of the perfusion assay. Data represent mean ± SE of at least four independent experiments.

**Figure 3:**
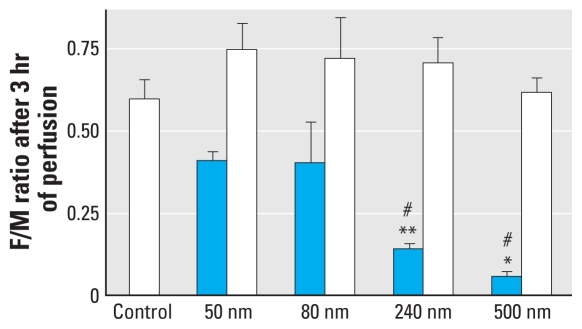
Size-dependent barrier capacity of the placental tissue. The ratios between fetal (F) and maternal (M) concentrations of ^14^C-antipyrine (open bars) and PS beads (blue bars) were calculated after 180 min of perfusion. The ^14^C-antipyrine values remain unchanged, whereas the perfusion rate of the beads showed size dependence. Data represent mean ± SE of at least four independent experiments. *p < 0.05 compared with 240-nm ratio value; **p < 0.05 compared with 80-nm ratio value; ^#^*p* < 0.05 compared with 50-nm ratio value.

Another important control was to assess the quality of the placental explant, which we achieved by monitoring the diffusion of ^14^C-antipyrine, which is known to rapidly cross the placenta and reach equal concentration in the maternal and fetal circuit after 4–6 hr (Challier 1985) (Figure 2B). If we started the perfusion with 25 μg/mL 50-nm PS beads in the maternal circuit, the level of particles measured after 180 min was 8.90 ± 1.80 μg/mL in the fetal perfusion medium and 18.1 ± 5.64 μg/mL in the maternal perfusion medium (Figure 2A). Because these concentrations remained stable thereafter, we performed a second administration of the same amount of beads to test whether the number of beads in the fetal circuit could be further increased. Similar to the first injection of PS beads, we observed a gradual decrease of the particles in the maternal perfusion medium but detected no significant increase of beads in the fetal perfusion medium. Administration of 80-nm particles resulted in a perfusion profile similar to the one for 50-nm beads, and the concentration of 80-nm particles measured in the fetal circulation after 180 min was 7.47 ± 1.77 μg/mL. After perfusion with 240-nm PS particles for 180 min, the bead concentration on the fetal side had dropped to 2.03 ± 0.29 μg/mL. Using 500-nm PS beads, nearly all particles were retained in the maternal circulation or placental tissue, and the concentration of beads detected in the fetal circulation was 0.31 ± 0.21 μg/mL (Figure 2A). The level of particles was low most likely because the 500-nm bead suspension was not monodisperse but rather contained particles < 500 nm in diameter (Figure 1D). We used the ratio of fetal-to-maternal concentration of PS beads detected after 180 min to estimate the amount of PS particles that crossed the placenta (Figure 3). These measurements showed that the translocation of PS beads across the placenta is clearly size dependent, and particles larger than 240 nm hardly pass this tissue barrier. The presence of PS beads did not affect the diffusion kinetic of ^14^C-antipyrine (Figures 2B, and 3), indicating that PS particles did not alter the transfer properties of the placental tissue.

At the end of each perfusion experiment, we took samples from the fetal circuit to check for the presence of beads. TEM micrographs indeed showed beads with diameters of up to 240 nm in the fetal perfusion medium (data not shown). This confirms the results of the fluorescence measurements that beads of around 240 nm were able to cross the human placenta.

### Viability of placental tissue after perfusion

**Figure 4:**
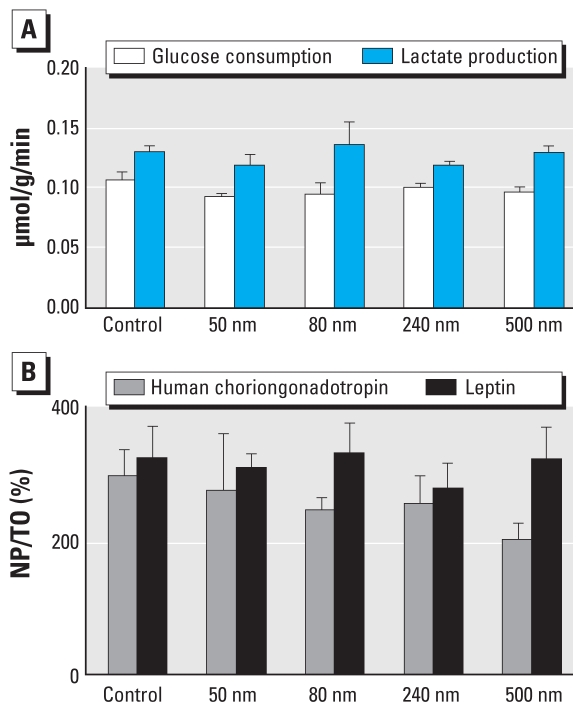
Viability and functionality of the placenta were not affected after perfusion with PS beads. (*A*) Glucose consumption and lactate production in the perfused tissue. (*B*) Human choriongonadotropin and leptin hormone net production during perfusion [NP divided by initial tissue content (T0)]. Data represent mean ± SE of at least four independent experiments.

The presence of PS beads in the placental tissue did not affect the viability of the placental explant, because the consumption of glucose and the production of lactate, human chorionic gonadotropin, and leptin were not altered (Figure 4). In addition, structural analysis of the explant after perfusion showed no alterations of the tissue, cells, or subcellular structures (data not shown).

## Discussion

The main findings of this study are that PS beads up to a diameter of 240 nm were taken up by the placenta and, further, were able to cross the placental barrier without affecting the viability of the explant. We used fluorescently labeled negatively charged PS beads of 50, 80, 240, and 500 nm as model particles because of their known biocompatibility and easy detection. This study provides evidence that the translocation of these beads across the placenta is size dependent.

We applied the particles at a single dose and at a high concentration, which can be assumed to be more representative of an intravenous injection, as reported for glioblastoma treatment with magnetic nanoparticles (Thiesen and Jordan 2008), than an environmental exposure scenario. With the *ex vivo* perfusion model, the mechanism of nanoparticle transport through the placenta very close to the *in vivo* situation can be studied, but it is limited to a perfusion period of a few hours: Chronic treatments with low doses over a long period are not possible because of tissue degradation (Panigel et al. 1967; Schneider et al. 1972). A further limitation of the model is that the perfusion rate measured in the explant represents only the late phase of pregnancy, for which the thickness of the barrier layer between the maternal and the fetal circuit is reduced and the number of fetal capillaries is increased (Enders and Blankenship 1999). Thus, the perfusion rates obtained with the *ex vivo* perfusion model may be higher than during the greater part of pregnancy *in vivo*.

The consideration of the mass concentration of nanoparticles is often criticized, and instead surface concentration or particle number concentration are suggested as a model (Stoeger et al. 2006). In the present study we intentionally selected a highly biocompatible nanomaterial to specifically examine the barrier capacity of the placenta without inducing toxic side effects. Thus, in this particular case, particle surface–dependent or mass-dependent toxicities play minor roles. Nevertheless, all relevant particle characteristics are summarized in Table 1 for later comparison and interpretation.

Possible transport routes for nanoparticles across the placenta are diffusion, vesicular transport, transmembranal transporter proteins, and the transtrophoblastic channel system. The perfusion profiles obtained for the 50- and 80-nm beads could be interpreted to suggest that these particles cross the placenta very quickly by simple diffusion. If so, the second application of PS beads should lead to a continuous translocation of the beads, which is not the case. The high doses applied could have induced dramatic clogging of the intervillous space on the maternal side, such that transfer routes were nearly blocked. Vesicle transport such as clathrin- or caveolin-mediated uptake is likely for the smaller PS particles because of their restricted size of 60 nm for clathrin-coated vesicles and 120 nm for caveolin-coated vesicles but rather questionable for bigger spheres of 240 nm (for comprehensive reviews, see Conner and Schmid 2003; Hillaireau and Couvreur 2009). The extent to which placenta transporter proteins in the basal- or brush-border membrane (e.g., of the syncytiotrophoblast) are involved in the nanoparticle transport remains unclear. The transtrophoblastic channel system represents a continuous membrane-lined tubular structure of about 20 nm in diameter that connects the intervillous space with the stroma and may act as a pressure-dependent valve regulating fetal water balance (Kertschanska et al. 1997). If the arterial pressure exceeds 80 cm H_2_O (corresponding to around 60 mmHg), this channel system is dilated, allowing the transport of liquid and small molecules. Whether this is true for the PS beads in our perfusion experiments requires further detailed investigations.

The critical size for PS nanoparticles to cross a placental barrier seems to be consistent with observations made at the air–blood barrier, where fluorescent PS beads of around 200 nm diameter penetrated cells in an energy-independent way (Geiser et al. 2005). But the capability of nanomaterials to cross the placenta appears to depend not only on particle size but also on the material composition or surface coating. It has been shown that polyethylene glycol–coated gold particles up to 30 nm in diameter were not able to cross the placenta with the same experimental setup as used in the present study (Myllynen et al. 2008). The fact that PS beads and gold nanoparticles interact differently with biological compounds suggests that, in general, the capability for transplacental transfer mechanism(s) has to be assessed separately for each type of nanoparticle.

## Conclusion

Our study showed a clear size-dependent barrier capacity of a healthy human placenta for PS nanoparticles, including an analysis of the viability and functionality of the tissue after the perfusion. We suggest that nanoparticles, in general, therefore have the potential for transplacental transfer. This potential underscores the need for further nanotoxicologic studies on this important organ system.

## Figures and Tables

**Table 1:** Summary of PS bead characteristics.

	PS beads			
Characteristic	50 nm	80 nm	240 nm	500 nm
Diameter (nm)^a^	49	83	240	504
Diameter (nm)^b^	43 ± 35	81 ± 44	220 ± 60	370 ± 96
Surface area (nm^2^)^c^	1.88E+03	5.41E+03	4.52E+04	2.00E+05
Internal volume (nm^3^)^c^	6.16E+04	2.99E+05	7.24E+06	6.70E+07
Density of PS (mg/nm^3^)^d^	1.05E–18	1.05E–18	1.05E–18	1.05E–18
Mass of one PS particle (mg)^c^	6.47E–14	3.14E–13	7.60E–12	7.04E–11
Initial PS particles/mL PM^c^	3.87E+11	7.95E+10	3.29E+09	3.55E+08
PS particle surface/mL PM (nm^2^)^c^	7.28E+14	4.30E+14	1.49E+14	7.10E+13
Detection limit of PS (μg/mL)^b^	< 1.25	< 1.64	< 0.63	< 0.21
Detection limit of PS (no./mL)^c^	1.94E+10	5.22E+09	8.22E+07	2.96E+06
Zeta potential in DD water (mV)^b^	−58.7 ± 2.26	−56.4 ± 2.12	−32.7 ± 0.78	−42.3 ± 0.49
Zeta potential in PM (mV)^b^	−6.23 ± 0.20	−7.40 ± 0.70	−8.91 ± 0.74	−12.8 ± 1.20

Abbreviations: DD, double distilled; PM, perfusion medium.

a According to manufacturer’s information.

b Experimentally determined (mean ± SE).

c Calculated values.

d From literature (Sharp and Beard 1950).
